# Supplementary material for: A Histone Deacetylase (HDAC) Inhibitor with Pleiotropic In Vitro Anti-Toxoplasma and Anti-Plasmodium Activities Controls Acute and Chronic Toxoplasma Infection in Mice
Source: Int J Mol Sci. 2022 Mar 17;23(6):3254. doi: 10.3390/ijms23063254 (PMC8952293; doi:10.3390/ijms23063254)
Supplement: Supplementary file 1 [file ijms-23-03254-s001.zip › ijms-1632862-supplementary.pdf]

Supplementary data S1

Scheme S1: **Synthetic routes of iodo-JF363.** Reagents and conditions: (a) m-OMe-p-I-Ph-COOH, DIEA, HOBt DCM; (b) KOH, MeOH/H_2O 40 °C; (c) (i) Cl-CO_2Et, NMM, DMF, (ii) NH_2OH, MeOH.

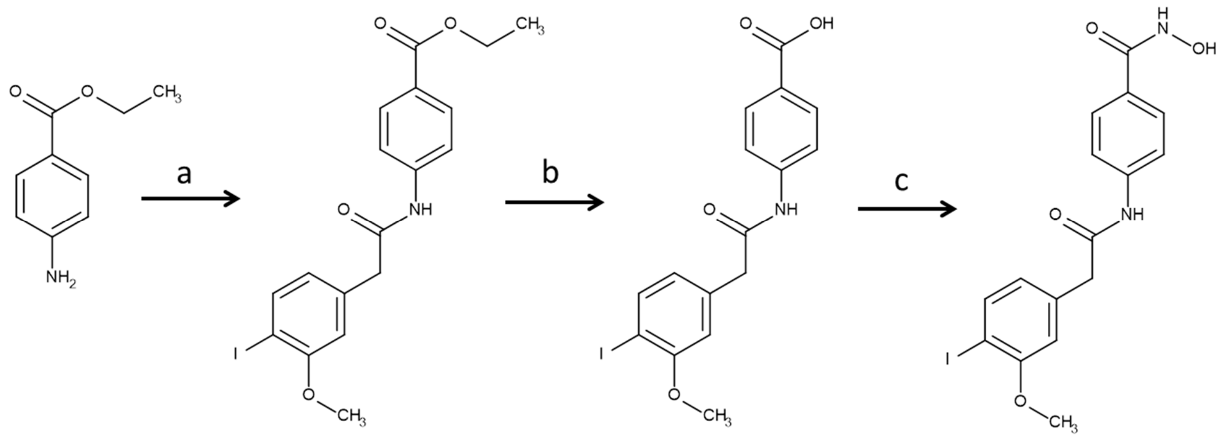

N-hydroxy-4-[2-(3-methoxy-4-iodophenyl)acetamido]benzamide (iodo-JF363)

^{1}H NMR (DMSO-d6, 300 MHz): d 11.09 (br s, 1H); 10.39 (s, 1H); 8.98 (br s, 1H); 7.73-7.66 (m, 3H); 7.64 (d, *J*=8.7Hz, 2H); 6.99 (d, *J*=1.5Hz, 1H); 6.73 (dd, *J*=1.6 and 7.9Hz, 1H); 3.82 (s, 3H); 3.66 (s, 2H). HPLC purity 96.2%. MS ESI+H^+ calc. 427.2 exp. 427.3

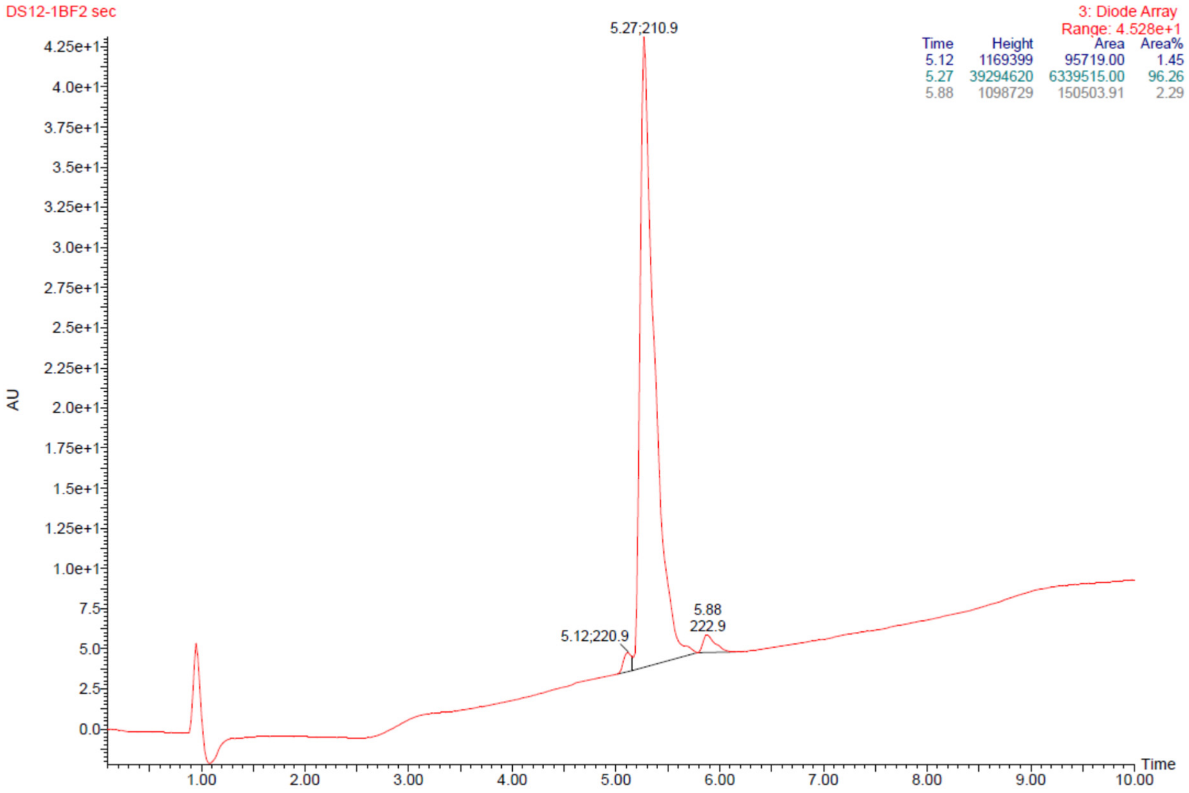

High Pressure Liquid Chromatography (HPLC) of iodo-JF**363**

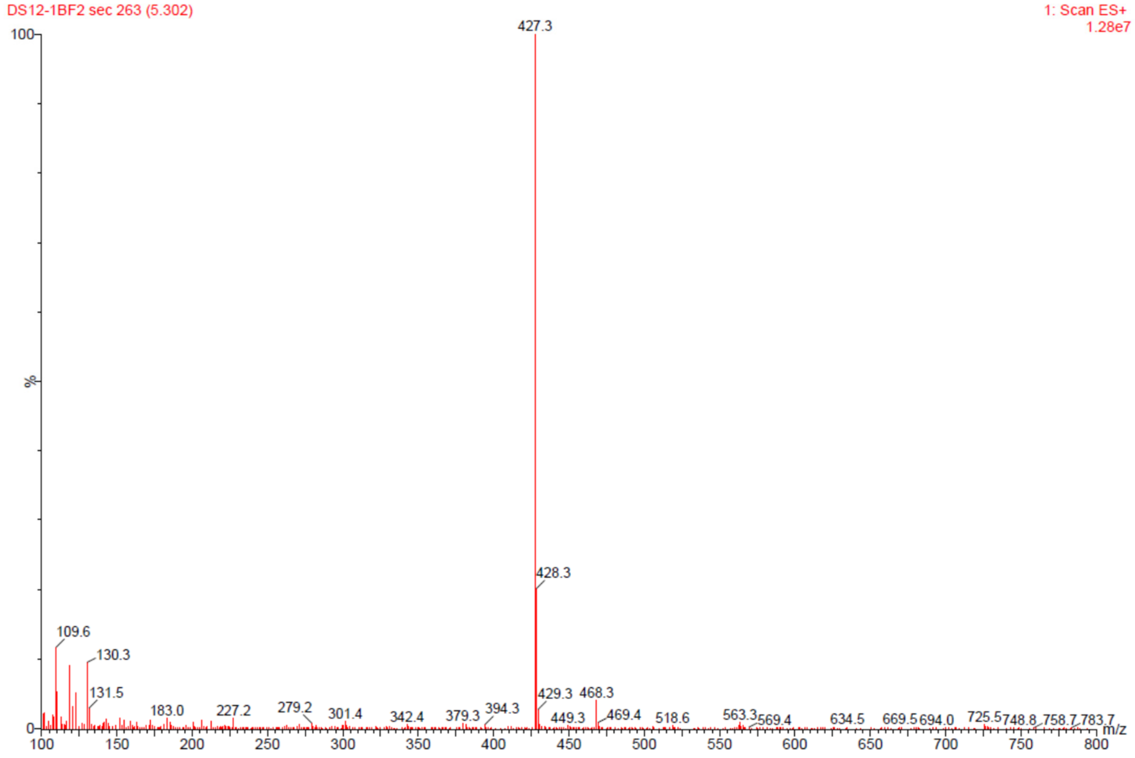

Mass Spectrometry (MS) of iodo-JF**363**

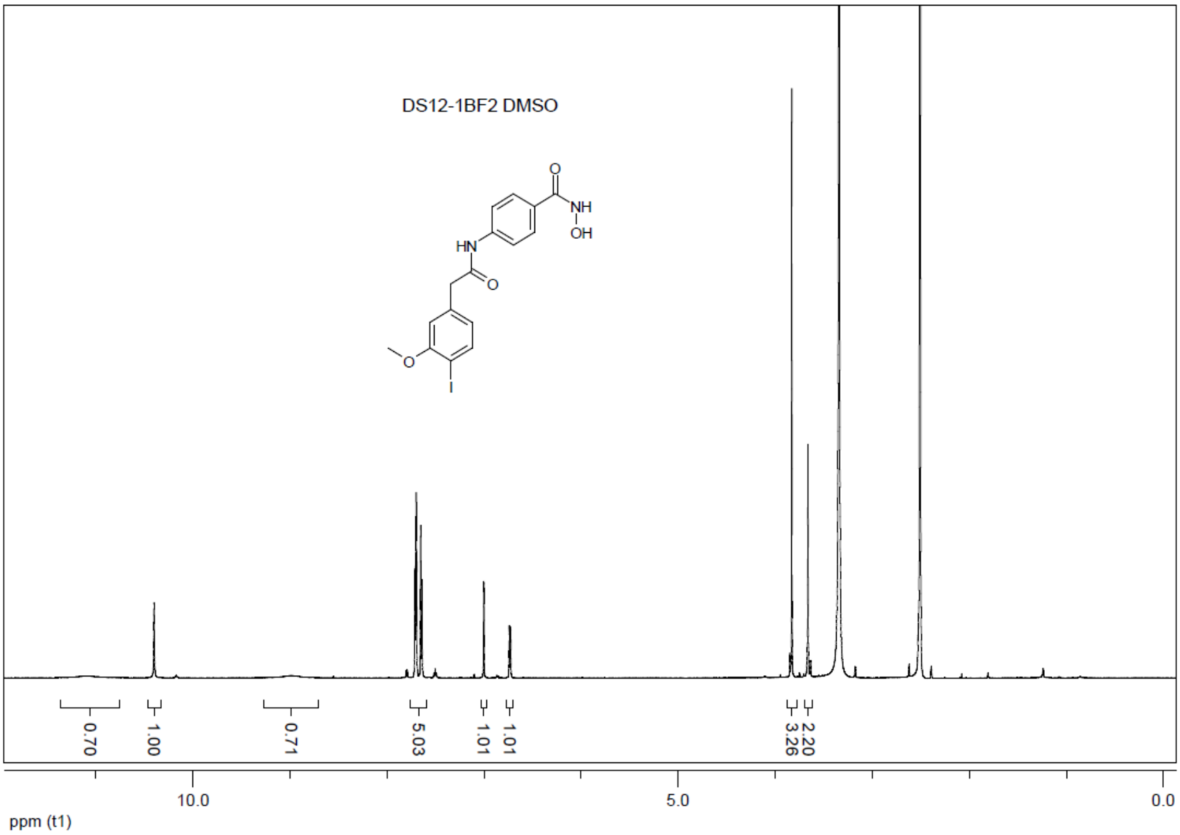

Nuclear Magnetic Resonance (NMR) of iodo-JF**363**
